# PTEN loss detection in prostate cancer: comparison of PTEN immunohistochemistry and PTEN FISH in a large retrospective prostatectomy cohort

**DOI:** 10.18632/oncotarget.19217

**Published:** 2017-07-10

**Authors:** Tamara L. Lotan, Asmus Heumann, Sebastian Dwertmann Rico, Jessica Hicks, Kristen Lecksell, Christina Koop, Guido Sauter, Thorsten Schlomm, Ronald Simon

**Affiliations:** ^1^ Pathology, Johns Hopkins School of Medicine, Baltimore, MD, USA; ^2^ Oncology, Johns Hopkins School of Medicine, Baltimore, MD, USA; ^3^ Institute of Pathology, University Medical Center Hamburg-Eppendorf, Hamburg, Germany; ^4^ Martini-Klinik Prostate Cancer Center, University Medical Center Hamburg-Eppendorf, Hamburg, Germany; ^5^ Department of Urology, University Medical Center Hamburg-Eppendorf, Hamburg, Germany

**Keywords:** prostatic carcinoma, PTEN, fluorescence in situ hybridization, immunohistochemistry, biomarker

## Abstract

*PTEN* deletion is an established prognostic biomarker in prostate cancer. We compared PTEN immunohistochemistry (IHC) and *PTEN* fluorescence *in situ* hybridization (FISH) in the largest existing radical prostatectomy cohort with clinical follow-up data. There was high concordance between IHC and FISH: 93% (3098/3330) of tumors with intact PTEN IHC showed absence of *PTEN* gene deletion and 66% (720/1087) of cases with PTEN protein loss by IHC showed *PTEN* gene deletion by FISH. 84% (447/533) of cases with *PTEN* homozygous gene deletion had PTEN protein loss by IHC. PTEN loss by IHC was associated with reduced PSA recurrence-free survival (RFS) in multivariable models (HR=1.3; 95% CI: 1.16-1.47). Among cases with either *PTEN* deletion or absence of *PTEN* deletion by FISH, PTEN loss by IHC was strongly associated with reduced RFS on univariable analysis (p=0.0005 and p<0.0001 respectively). Among cases with intact PTEN by IHC, homozygous (p=0.04) but not heterozygous (p=0.10) *PTEN* gene deletion was weakly associated with reduced RFS. Among cases with PTEN loss by IHC, both homozygous (p=0.0044) and heterozygous (p=0.0017) *PTEN* gene deletion were associated with reduced RFS. These data support the utility of PTEN IHC and *PTEN* FISH as complementary screening tools for PTEN loss in prostate cancer.

## INTRODUCTION

With increasing numbers of patients deferring definitive therapy for prostate cancer in favor of active surveillance, there is an unmet need for molecular biomarkers that help to distinguish indolent and lethal prostate tumors. *PTEN* gene deletion remains one of the few common genomic alterations in prostate cancer that is reproducibly associated with poor outcomes [1–13]. Because PTEN loss is commonly focal and subclonal in primary prostate tumors [4, 14–16], *in situ* methodologies for PTEN loss detection may be preferable to methods that assess copy number variation based on nucleic acid extraction, such as sequencing. Immunohistochemistry (IHC) and fluorescence *in situ* hybridization (FISH) have both been used to assess for PTEN loss in formalin fixed paraffin embedded (FFPE) tissues. Of the two, IHC-based detection of PTEN loss in prostate cancer is less expensive and less time-consuming for the routine screening of prostate tumor specimens, and may be easier to adapt to the current pathology work flow for risk assessment in prostate cancer. In cases of focal loss, detection of *PTEN* gene deletion by FISH can be especially challenging and more easily accomplished by IHC. Finally, in addition to genomic deletion, PTEN protein levels may be altered by small insertions/deletions or point mutations in the gene or even by microRNA- or epigenetic-regulated mechanisms which would not be detectable by FISH [4, 17–19]. However, only a few studies have directly compared PTEN IHC and *PTEN* FISH in large cohorts of prostate tumors with clinical outcome information [4, 17, 18, 20–24].

We previously optimized and genetically validated a PTEN IHC assay for the detection of PTEN loss in prostate cancer specimens [4, 23, 24]. Using this assay, we showed that PTEN loss is associated with *PTEN* gene deletion [24], and independently associated with an increased risk of biochemical recurrence [7, 25] and lethal prostate cancer [13] in several large, multi-institutional cohorts of patients largely treated by radical prostatectomy. Similarly, *PTEN* loss by FISH has been reported to be associated with an increased risk of biochemical recurrence in a large cohort of patients treated by radical prostatectomy at the University Medical Center Hamburg-Eppendorf, Hamburg, Germany [5, 8]. Here, in the largest cohort ever studied by both techniques, we evaluated the performance of PTEN IHC in the Hamburg cohort and compared it to previously reported *PTEN* FISH results. We demonstrate that PTEN IHC and FISH results are largely concordant and associated with a similar increase in risk of biochemical recurrence in multivariable models. Finally, taking advantage of the relatively large cohort to examine the clinical outcomes of cases with discordant results by IHC and FISH, we find that these methods may provide complementary information in a subset of cases.

## RESULTS

**Figure 1:**
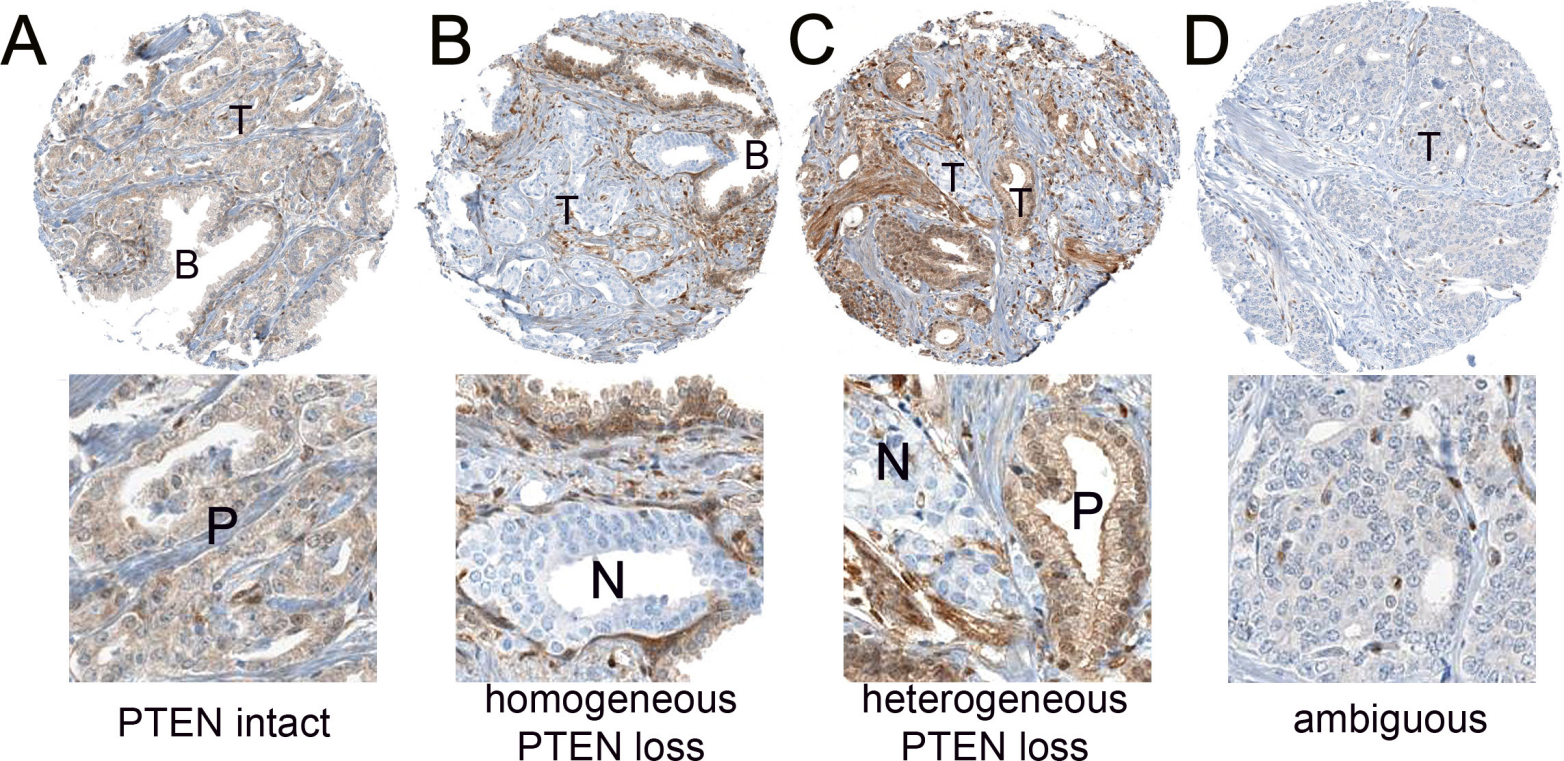
Representative PTEN immunohistochemistry results **(A)** PTEN intact in tumor cells (T), with equivalent staining in nearby benign glands **(B)**. Higher magnification inset below shows positive (P) staining tumor glands. (B) PTEN homogeneous loss in tumor glands (T), with intact staining in nearby benign glands (B) and stroma. Higher magnification inset below shows negative (N) staining tumor glands. **(C)** PTEN heterogeneous loss, with staining loss in some but not all sampled tumor cells (T). Higher magnification inset below shows positive (P) staining and negative (N) staining tumor glands. **(D)** PTEN ambiguous staining. PTEN is decreased but not lost in tumor glands and absence of background benign glands for comparison makes this case difficult to interpret. Higher magnification inset below shows glands with ambiguous PTEN immunostaining.

PTEN IHC was initially assessed in a total of 9033 tumors for the current study, of which 22% (n=2005) showed any PTEN loss (including 20% or n=1794 with homogeneous PTEN loss and 2% or n=211 with heterogeneous loss), 67% (n=6075) showed intact PTEN protein and 11% (953) showed ambiguous PTEN IHC (Figure 1). Of these cases, 52% (4732/9033) had evaluable *PTEN* FISH data available from previous studies, and FISH images from the tumors from the current study are available in these published manuscripts [5, 8]. Of these, 23% (1087/4732) showed any PTEN loss by IHC, including 20% (966/4732) with homogeneous PTEN loss and 3% (121/4732) with heterogeneous loss. An additional 70% (3330/4732) showed intact PTEN protein by IHC and 7% (315/4732) showed ambiguous PTEN IHC results. The remainder of the manuscript will focus on the subset of cases with evaluable IHC and FISH results (n=4417). The rates of *PTEN* gene and PTEN protein loss were quite similar in the subset with both FISH and IHC results compared to the entire evaluable cohort with interpretable results for each assay reported separately (Table 1). Among this subset of cases, PTEN IHC loss was found in 25% (1087/4417) of cases, including 22% with homogeneous PTEN loss and 3% with heterogeneous PTEN loss. The remaining 75% (3330/4417) had intact PTEN protein. Among these cases with IHC and FISH interpretable results, *PTEN* gene deletions were found in 21% (952/4417) of cases, including 12% (533/4417) with homozygous gene deletion and 9% (419/4417) with heterozygous gene deletion. The remaining 79% (3465/4417) had normal *PTEN* by FISH, similar to what was reported previously [5, 8].

**Table 1:** Comparison of PTEN IHC and *PTEN* FISH results across all cases with available data

n (%)	ambiguous IHC	PTEN IHC intact	PTEN IHC loss heterogeneous	PTEN IHC loss homogeneous
***PTEN* FISH normal**	280 (8%)	3098 (83%)	74 (2%)	293 (8%)
***PTEN* FISH heterozygous deletion**	18 (4%)	146 (33%)	23 (5%)	250 (57%)
***PTEN* FISH homozygous deletion**	17 (3%)	86 (16%)	24 (4%)	423 (77%)

Overall, there was a high concordance between PTEN IHC and FISH (p<0.0001). 93% (3098/3330) of tumors with intact PTEN IHC showed absence of *PTEN* gene deletion and 66% (720/1087) of cases with PTEN protein loss by IHC showed *PTEN* gene deletion by FISH. Similarly, 89% (3098/3465) of tumors with normal *PTEN* by FISH showed intact PTEN IHC and 76% (720/952) of cases with *PTEN* gene deletion by FISH showed PTEN protein loss by IHC. Overall, 84% (447/533) of cases with *PTEN* homozygous gene deletion had PTEN protein loss by IHC. 65% (273/419) of tumors with *PTEN* heterozygous gene deletion showed PTEN protein loss by IHC. Of the discordant cases with PTEN loss by IHC and normal PTEN FISH results, 20% showed heterogeneous PTEN loss. Notably, 20% (74/367) of the discordant cases (loss of PTEN protein expression by IHC and normal *PTEN* by FISH analysis) showed heterogeneous PTEN protein loss in some, but not all, sampled tumor glands, compared to only 11% (121/1087) of cases with PTEN IHC loss overall which showed heterogeneous PTEN loss. This suggests the possibility that tumor heterogeneity could explain at least some of the discordant results.

The negative predictive value for intact PTEN IHC was 93% (3098/3330) for lack of any gene deletion and 97% (3244/3330) for lack of homozygous *PTEN* deletion. The positive predictive value of PTEN IHC loss for presence of any *PTEN* gene deletion (homozygous or heterozygous) was 66% (720/1087) overall, or 70% (673/966) for homogeneous PTEN protein loss and 39% (47/121) for heterogeneous PTEN protein loss.

**Figure 2:**
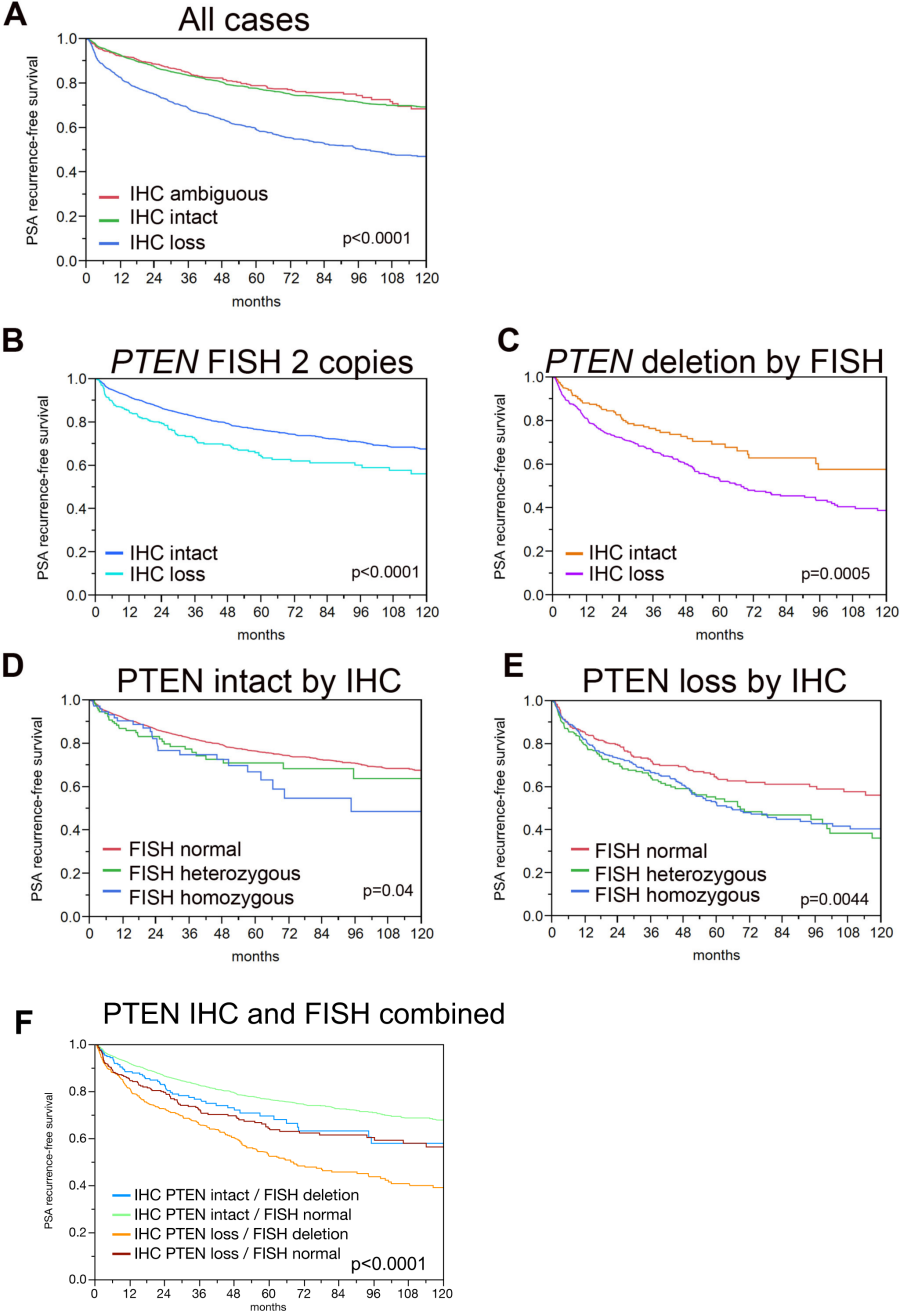
Kaplan-Meier analysis for PSA recurrence-free survival by PTEN IHC and FISH status **(A)** All cases with PTEN IHC and FISH results, stratified by PTEN IHC intact (n=2,970, n_censored_=2,372) and PTEN IHC loss (n=992, n_censored_=615). **(B)** All cases with normal *PTEN* FISH results, stratified by PTEN IHC intact (n=2,756 n_censored_=2214) and PTEN IHC loss (n=332, n_censored_=229). **(C)** All cases with *PTEN* deletion by FISH stratified by PTEN IHC intact (n=214, n_censored_=158) and PTEN IHC loss (n=660, n_censored_=386). **(D)** All cases with PTEN intact by IHC, stratified by *PTEN* FISH normal (n=2,756, n_censored_=2,214), *PTEN* FISH heterozygous deletion (n=137, n_censored_=104) and *PTEN* FISH homozygous deletion (n=77, n_censored_=54). For comparison between *PTEN* normal and *PTEN* heterozygous deletion, p=0.1016. For comparison between *PTEN* normal and *PTEN* homozygous deletion, p=0.0393. For comparison between *PTEN* homozygous deletion and *PTEN* heterozygous deletion, p=0.5776. **(E)** All cases with PTEN loss by IHC, stratified by *PTEN* FISH heterozygous deletion (n=248 n_censored_=144) and *PTEN* FISH homozygous deletion (n=412, n_censored_=242). For comparison between *PTEN* normal and *PTEN* heterozygous deletion, p=0.0017. For comparison between *PTEN* normal and *PTEN* homozygous deletion, p=0.0044. For comparison between *PTEN* homozygous deletion and *PTEN* heterozygous deletion, p=0.4777.

Associations between PTEN status and clinical-pathologic variables are shown in Table 2. PTEN loss by IHC was associated with a number of clinical-pathologic parameters (Table 2) and similar results are available for PTEN FISH in the same cohort in a previously published manuscript [5]. Increasing frequency of PTEN loss was seen in association with increasing pathologic stage (p<0.0001), increasing Gleason score (p<0.0001), presence of lymph node metastases (p<0.0001), higher pre-operative PSA levels (p<0.0001) and with a higher frequency of positive surgical margins (p<0.0001). Accordingly, patients with PTEN IHC loss had decreased PSA recurrence-free survival compared to patients with intact or ambiguous PTEN IHC status in univariable analyses (p<0.0001, Figure 2A). On multivariable analysis including pre-operative PSA level, pathologic tumor stage, Gleason score, lymph node status and margin status, PTEN loss by IHC remained significantly associated with decreased PSA recurrence-free survival, with a hazard ratio of 1.3 (95% CI:1.16-1.47), a hazard ratio roughly equivalent to that seen for positive lymph node status (Table 3).

**Table 2:** Associations between PTEN IHC status and clinical-pathologic variables

	n evaluable	% PTEN IHC intact	% PTEN IHC loss	p value
**All cancers**	7,813	75.7	24.2	
**Tumor stage**				
pT2	4,675	84.8	15.2	
pT3a	1,917	68.2	31.9	<0.0001
pT3b-pT4	1,181	52.5	47.4	
**Gleason grade**				
≤3+3	1,325	85.9	14	
3+4	4,529	78.7	21.2	
4+3	1,445	62.5	37.5	<0.0001
≥4+4	467	59.3	40.7	
**Gleason grade quantification**				
≤3+3	1,244	85.1	14.9	
3+4 (≤5% pattern 4)	952	86.5	13.4	
3+4 (6-10% pattern 4)	976	81.9	18	
3+4 (11-20% pattern 4)	814	74.7	25.3	
3+4 (21-30% pattern 4)	486	72.7	27.4	
3+4 (31-49% pattern 4)	379	69.4	30.6	
3+4 (Tertiary pattern 5)	259	75.3	24.7	<0.0001
4+3 (50-60% pattern 4)	325	63.7	36.3	
4+3 (61-80% pattern 4)	305	62.9	37	
4+3 (>80% pattern 4)	71	67.6	32.4	
4+3 (Tertiary pattern 5)	425	61	39.1	
≥4+4	314	57	43	
**Lymph node metastasis**				
N0	4,794	75.3	24.7	
N+	592	51.3	48.6	<0.0001
**Preop. PSA level (ng/ml)**				
<4	903	70.9	29.1	
4-10	4,582	77.4	22.6	
10-20	1,634	75.7	24.4	<0.0001
>20	603	72.5	27.5	
**Surgical margin**				
negative	6,019	78	22	
positive	1,619	68	32	<0.0001

**Table 3:** Multivariable analysis of association of PTEN IHC status with clinical-pathologic variables

		HR	95% CI	p-value
	4-10 vs <4	1.1	0.91-1.43	
**PSA level**	10-20 vs 4-10	1.3	1.12-1.45	<0.0001
	>20 vs 10-20	1.3	1.12-1.51	
	pT3a vs pT2	1.9	1.66-2.25	
**pT stage**	pT3b vs pT3a	1.5	1.28-1.69	<0.0001
	pT4 vs pT3b	1.3	0.91-1.76	
	3+4 vs ≤3+3	2.2	1.67-2.81	
**pGleason**	4+3 vs 3+4	2	1.8-2.32	<0.0001
	≥4+4 vs 4+3	1.2	1-1.4	
**pN Stage**	N+ vs N0	1.4	1.21-1.65	<0.0001
**margin status**	positive vs negative	1.2	1.03-1.32	0.0164
**PTEN**	loss vs intact	1.3	1.16-1.47	<0.0001

We performed ROC analyses using PSA recurrence (biochemical recurrence, BCR) as the categorical response to estimate whether addition of PTEN IHC and/or FISH can improve the predictive power beyond established prognostic parameters including pT stage, Gleason grade, nodal stage and pre-surgical PSA value as the basic model. Addition of PTEN IHC increased the area under the ROC curve (AUC) from 0.701 to 0.741, and addition of PTEN FISH increased the AUC from 0.701 to 0.745. If both PTEN IHC and FISH were added to the basic model, there was only a slight further increase to 0.749.

Next, we examined the association of PTEN loss by IHC in combination with the *PTEN* FISH results with outcomes in univariable analysis. PTEN loss by IHC was associated with decreased PSA recurrence-free survival among tumors with *PTEN* gene deletion and among tumors with normal PTEN by FISH (p=0.0005 and p<0.0001 respectively) (Figure 2B, 2C). Conversely, among cases with intact PTEN by IHC, homozygous (p=0.04) but not heterozygous (p=0.10) *PTEN* gene deletion was weakly associated with decreased PSA recurrence-free survival compared to cases with *PTEN* FISH normal (Figure 2D). However among cases with PTEN loss by IHC, both homozygous (p=0.0044) and heterozygous (p=0.0017) *PTEN* gene deletion were associated with decreased PSA recurrence-free survival compared to cases with *PTEN* FISH normal (Figure 2E). The combined impact of the IHC and FISH findings on patient prognosis are shown in Figure 2F. The best prognosis was found for cancers with intact PTEN protein and normal PTEN copy numbers, while cancer with PTEN protein loss (IHC) and deletion (FISH) had the worst outcome (p<0.0001). An intermediate prognosis was found for tumors harboring only one alteration (i.e., PTEN protein loss or deletion). There was no difference between cancers with intact PTEN protein but deletion by FISH or loss of PTEN protein but normal copy numbers (p=0.4174), but both intermediate groups were significantly different from cancers with concordant lack or presence of PTEN alterations (p≤0.0125 each).

## DISCUSSION

There is an increasing need for tissue-based prognostic biomarkers in prostate cancer with growing numbers of patients being enrolled on active surveillance protocols rather than undergoing definitive therapy. Loss of the *PTEN* tumor suppressor gene is arguably one of the most reproducible and best validated genetic biomarkers in prostate cancer. Indeed, its loss has been associated with increased risk of prostate cancer recurrence [1, 5, 7, 8, 12, 25] and death [9, 13, 26] in numerous independent studies. Because *PTEN* is most commonly inactivated in prostate cancer by large scale genomic deletion, *PTEN* FISH has historically been used to query *PTEN* status in tissue samples. However, interphase FISH is relatively expensive and time-consuming to perform, requiring the detailed enumeration of fluorescent signals in 20-100 nuclei under oil-immersion microscopy.

To address these challenges, several groups have developed immunohistochemistry (IHC) assays to query PTEN status in archived formalin fixed tissue samples [4, 27, 28]. While a number of such assays have been published, for the most part, these assays have largely been compared to PTEN FISH in only small scale studies with less than 200 samples [4, 17, 18, 20–24]. In the few larger studies that compared IHC and FISH, including one previous study on the cohort described in the current study, there was no significant [5] or only weak [9] correlation between the assays, likely due to failure of the IHC assay to validate.

To improve on existing assays, we used a commercially available rabbit monoclonal antibody against PTEN coupled with an automated staining system to develop and validate a clinical-grade PTEN IHC test. Our simple scoring system for this assay has shown high inter-observer reproducibility in a number of cohorts, with κ values exceeding 0.9 [13, 25]. Using this assay, our group previously demonstrated that PTEN protein loss is associated with an increased risk of recurrence and progression in surgically treated cohorts of prostate cancer patients [6, 7, 25]. More importantly, PTEN loss using our assay is independently associated with increased risk of lethal prostate cancer in an independent, large population-based cohort [13]. More recently, we have examined the correlation between a 4-color FISH assay and PTEN IHC results in the Canary Retrospective Tissue Microarray cohort and found excellent concordance [24]. However this previous study included only a small number of cases with discordant status by FISH and IHC which precluded any meaningful analysis of outcomes in this group.

In the current study, we compared PTEN IHC and FISH results across the largest available radical prostatectomy cohort with clinical follow-up. Successful analysis of more than 9000 tumors with PTEN IHC and more than 6700 tumors with PTEN FISH resulted in more than 4400 tumors with concurrent PTEN FISH and IHC results for comparison. Overall, there was an excellent concordance between the two techniques. Reasons for discordance between PTEN IHC and FISH likely include tumor heterogeneity [14, 16, 29]. Because IHC and FISH analysis were performed several years apart, the TMA sections used for each were not adjacent to one another and may, thus, represent different subclones of the same tumor in a fraction of cases, a weakness of the current study design that was unavoidable for logistical reasons. Intratumoral heterogeneity in PTEN loss can be seen in upwards of 50% of primary prostate tumors [13, 14, 16] and might be one important explanation for the 15% of cases with homozygous *PTEN* deletion by FISH that had intact PTEN by IHC. Among cases with normal or hemizygous *PTEN* by FISH yet PTEN loss by IHC, another explanation could be the occurrence of alterations that are undetectable by our FISH assay yet lead to protein loss, such as truncating mutations in *PTEN* (nonsense, frameshift and splice site mutations), structural rearrangements, epigenetic alterations or modifications influencing protein stability and half-life [16, 30–33]. Finally, it cannot be excluded that a fraction of samples were misclassified due to technical reasons. FISH scoring cut-offs are determined by studying a relatively small number of cases with known *PTEN* genomic status, however because of the statistical nature of these cut-off determinations, a small fraction of cases may be misclassified by FISH [34]. Likewise, IHC can only detect protein concentrations beyond the detection limit of IHC, and the amount of detectable protein may be influenced by many non-biological factors such as tissue quality, protein preservation, antibody concentration and antigen retrieval efficacy [35, 36].

Though we have conducted smaller comparisons of our IHC assay with FISH previously [24], the large number of cases in the current study enabled us to examine the clinical significance of discordance between PTEN IHC and FISH. Given that many factors other than the gene copy number can influence protein levels, it seems unlikely that discrepant IHC/FISH findings are merely due to failure of one of the two tests. The fact that the presence of one alteration (either on the DNA or on the protein level) is associated with a significant worsening of the patient’s prognosis, and that the prognostic impact of isolated protein or DNA alterations is virtually identical but less severe compared to when both types of alterations co-occur, may fit with the proposed dose-dependency of PTEN’s tumor suppressor function [37]. Our data thus suggest that PTEN protein loss and gene deletion represent complementary mechanisms of PTEN inactivation and each provides complementary prognostic information. In fact, multivariate ROC-AUC modelling including PTEN IHC and PTEN FISH in addition to established prognostic parameters such as pT stage, Gleason score, nodal stage and pre-surgical PSA indicates that both IHC and FISH similarly improve predictive accuracy. That PTEN loss by IHC identified cancers with markedly reduced PSA recurrence-free survival independently of the FISH status is consistent with the idea that a “direct” analysis of the protein – i.e., the “active” component of a gene - may be superior to “indirect” analysis of its mere copy number state. However, that FISH analysis also predicted prognosis in the subset of cancers with PTEN loss by IHC, demonstrates that copy number analysis may hold relevant prognostic potential beyond the protein level. A possible explanation is that genomic deletion can also be a surrogate marker for genetic instability, which is generally linked to poor outcome [38]. Profiling studies using next generation sequencing have shown that prostate cancers often harbor multiple additional structural genomic alterations [31, 32, 38, 39], and we have demonstrated earlier in the same cohort as used in the present study that many of these deletions are linked to poor outcome [5, 40–44]. Finally, we also cannot exclude the possibility that since the FISH and IHC assays were not conducted on adjacent sections, performing both assays was a mechanism to evaluate the tumor for PTEN loss in two separate areas, identifying more cases with heterogeneous PTEN loss than either single test alone.

Irrespective of the reasons leading to discrepant IHC and FISH findings, the strong and independent prognostic impact of both methods suggests that re-testing with a second method could be justified in a subset of cases, particularly in cases with *PTEN* deletion or lack of deletion by FISH or PTEN protein loss by IHC. Although a negative result with one test had a high negative predictive value for the other method (IHC: 93%, FISH: 89%), these figures do also demonstrate that about 10% of all cancers (accounting for 45% of PTEN-deficient cases) harbor PTEN alterations that remain undetected if only one method is employed. Our data do not argue for a particular sequence of PTEN testing. Arguments in favor of first-line FISH are that FISH eliminates the need to compare staining intensities between cancer and normal cells and that it gives a clear-cut “yes/no” answer with regards to the genomic status of the tumor. Arguments for using IHC as a first test include the lower costs and shorter analysis time, the higher rate of technical failure with FISH, as well as the fact that the IHC test is more easily integrated into standard pathology laboratory work flow. IHC analysis could, therefore, be the method of choice in places where no FISH analysis is possible.

There are a number of limitations of the current study. Technical difficulties associated with optimizing FISH hybridization on TMA slides meant that more than half of the cases with PTEN IHC results did not have accompanying FISH results. Though we showed that the samples with data by both methodologies were essentially comparable to the larger set with IHC results, we cannot exclude some bias in the cases that were excluded for unavailable FISH results and thus not studied by both methods. In addition, the design of the TMA set in the current study sampled only one core of tumor tissue for each case. Tumor heterogeneity is a major factor in all TMA and prostate biopsy studies, and the one-core-per-cancer sampling strategy in our TMA is not suitable to address inter- or even intratumoral heterogeneity. The very low likelihood to have relevant heterogeneity among the about 500 tumor cells that are typically present in a 0.6 mm TMA spot is perfectly reflected by the markedly lower rate of heterogeneous PTEN IHC loss (2%) in the current study as compared to other TMA cohorts with 3-4 tumor cores sampled from each case [13, 25]. However, it is noteworthy, that the amount of tissue studied in a minute TMA spot closely resembles that of a typical core needle biopsy, making our one-spot-per-cancer approach a suitable surrogate for molecular analyses on diagnostic biopsies. Finally, the “true” PTEN status is unknown in cases with discordant FISH and IHC results. Though FISH can detect deletions resulting from genomic rearrangement that are the most common mechanism of loss in prostate cancer, it will miss very small structural variations, indels and missense mutations that have been found by next generation sequencing in up to 5% of cancers. In addition, IHC does not provide information about protein activity, and there is no established threshold to distinguish between “sufficient” and “insufficient” levels of the protein with respect to downstream oncogenic signaling.

In conclusion, in the largest radical prostatectomy cohort studied to date with clinical outcome information, PTEN loss by our clinical-grade IHC assay is highly concordant with *PTEN* gene status by FISH, and associated with poor outcomes in the disease. That a relevant fraction of about 10% cancers yield discrepant results between FISH and IHC analysis, which are similarly linked to tumor aggressiveness, suggests that clinically relevant PTEN alterations can be missed if only one method is applied. Our findings thus argue for a combination of both methods in order to obtain the most accurate information on PTEN status with current state-of–the-art diagnostic *in situ* methods.

## MATERIALS AND METHODS

### Subject selection and tissue microarray design

The features of this cohort have been previously described in detail elsewhere [5]. Briefly, the cohort consists of radical prostatectomy specimens from 13,665 consecutive patients undergoing radical prostatectomy between 1992 and 2008 at the Department of Urology, University Medical Center Hamburg-Eppendorf, Hamburg, Germany. PTEN immunohistochemistry (IHC) was assessed in a total of 9033 tumors for the current study and 4732 of these patients had *PTEN* fluorescence *in situ* hybridization (FISH) data available for comparison. Clinical follow-up data were available for 4203 cases. The median follow-up was 46.7 months (range, 1 to 219 months). None of the patients received neoadjuvant endocrine therapy. In all patients, PSA values were measured quarterly in the first year, followed by biannual measurements in the second year and annual measurements after the third year following surgery. Recurrence was defined as a postoperative PSA of 0.2 ng/mL, increasing thereafter. The first PSA value of 0.2 ng/mL or greater was used to define the time of recurrence. Salvage therapy was initiated in cases of biochemical relapse. Patients without evidence of tumor recurrence were censored at the last follow-up. All cancers were arrayed on 30 tissue microarrays blocks, where each tumor was sampled once, utilizing 0.6 mm cores. The area selected for sampling was guided not by Gleason grade, but to maximize tumor content available for analysis in the TMA core.

### Immunohistochemistry assays

PTEN immunohistochemistry (IHC) was performed as recently reported [13, 25]. Briefly, the protocol uses the Ventana automated staining platform (Ventana Discovery Ultra, Ventana Medical Systems, Tucson, AZ) and a rabbit anti-human PTEN antibody (Clone D4.3 XP; Cell Signaling Technologies, Danvers, MA).

### Immunohistochemistry scoring

After staining for PTEN, all TMAs were scanned at 20x magnification (Aperio, Leica Microsystems, Buffalo Grove, IL) and segmented into TMAJ for scoring (http://tmaj.pathology.jhmi.edu/). PTEN protein status was blindly and independently scored by a trained pathologist (TLL) using a previously validated scoring system (Figure 1). A tissue core was considered to have PTEN protein loss if the intensity of cytoplasmic and nuclear staining was markedly decreased or entirely negative across >10% of tumor cells compared to surrounding benign glands and/or stroma, which provide internal positive controls for PTEN protein expression [4, 13]. If the tumor core showed PTEN protein expressed in >90% of sampled tumor glands, the tumor was scored as PTEN intact. If PTEN was lost in <100% of the tumor cells sampled in a given core, the core was annotated as showing heterogeneous PTEN loss in some, but not all, cancer glands (focal loss). Alternatively, if the core showed PTEN loss in 100% of sampled tumor glands, the core was annotated as showing homogeneous PTEN loss. Finally, a small percentage of cores were scored as having ambiguous PTEN IHC results. This occurred when the intensity of the tumor cell staining was light or absent in the absence of evaluable internal benign glands or stromal staining.

### Initial blinded analysis of *PTEN* FISH

PTEN FISH was performed as previously described [5, 8]. Briefly, a dual-color FISH probe set was used consisting of two SpectrumGreen-labeled bacterial artificial chromosome clones (RP11-380G5 and RP11-813O3; Source Bioscience, Nottingham, UK) and a SpectrumOrange-labeled commercial centromere 10 probe (06J36-090; Abbott, Wiesbaden, Germany) as a reference. The predominant red and green signal numbers were recorded for each FISH probe. A total of 659 tissue spots were excluded from FISH analysis because basal cell marker 34βE12 analysis indicated lack of tumor cells. Thresholds for *PTEN* FISH analysis were established from 0.6-mm tissue spots from seven tumors with a known *PTEN* deletion (four with a heterozygous and three with a homozygous deletion), based on single-nucleotide polymorphism (SNP) array copy number analysis. In five of these tumors, *PTEN* signal losses by FISH were found in all analyzed tissue blocks. The two remaining cancers had tissue blocks with and without *PTEN* deletion, indicating the presence of intratumoral heterogeneity. In all seven cases, tumor blocks with *PTEN* deletion had FISH signal losses in most (at least 60%) tumor cells. According to these findings, homozygous deletion of *PTEN* was defined as complete absence of *PTEN* FISH probe signals in ≥60% of tumor nuclei of the tissue spot, with the presence of one or two *PTEN* FISH signals in adjacent normal cells. Tissue spots with a lack of *PTEN* signals in all (tumor and normal cells) or lack of any normal cells as an internal control for successful hybridization of the *PTEN* probe were excluded from analysis. Heterozygous deletion of *PTEN* was defined as the presence of fewer *PTEN* signals than centromere 10 probe signals in ≥60% tumor nuclei.

### Statistics

Statistical calculations were performed with JMP® 10.0.2 software (SAS Institute Inc., NC, USA). Contingency tables and the chi^2^-test were performed to search for associations between PTEN alterations and tumor phenotype. Survival curves were calculated according to Kaplan-Meier. The Log-Rank test was applied to detect significant differences between groups. Cox proportional hazards regression analysis was performed to test the statistical independence and significance between pathological, molecular and clinical variables. Logistic regression was used to quantify the area under the receiver-operator curve (ROC-AUC).
